# The meniscal extrusion index is a reliable indirect sign of different meniscal lesion patterns: a classification based on percentage of meniscal extrusion

**DOI:** 10.1007/s00167-023-07525-6

**Published:** 2023-08-31

**Authors:** Riccardo Compagnoni, Paolo Ferrua, Carlo Minoli, Raschid Fajury, Rossella Ravaglia, Alessandra Menon, Pietro Simone Randelli

**Affiliations:** 1 Department of Biomedical, Surgical and Dental Sciences, Università degli Studi di Milano, Via della Commenda, 10, 20122 Milan, Italy; 2 Laboratory of Applied Biomechanics, Department of Biomedical Sciences for Health, Università degli Studi di Milano, Via Mangiagalli 31, 20133 Milan, Italy; 3 U.O.C. 1° Clinica Ortopedica, ASST Gaetano Pini-CTO, Piazza Cardinal Ferrari 1, 20122 Milan, Italy; 4 REsearch Center for Adult and Pediatric Rheumatic Diseases (RECAP-RD), Department of Biomedical Sciences for Health, Università degli Studi di Milano, Via Mangiagalli 31, 20133 Milan, Italy; 5 Scuola di Specializzazione in Statistica Sanitaria e Biometria, Dipartimento di Scienze Cliniche e di Comunità, Università degli Studi di Milano, Milan, Italy; 6 U.O.C. Week Surgery, ASST Gaetano Pini-CTO, Piazza Cardinal Ferrari 1, 20122 Milan, Italy; 7 Università degli Studi di Milano, Via Festa del Perdono 7, 20122 Milan, Italy

**Keywords:** Knee, MRI, Meniscus, Extrusion, Root, Tear, Imaging, Arthroscopy, Classification, Meniscal Extrusion Index

## Abstract

**Purpose:**

This study's goal is to propose a straightforward classification system based on the MEI (Meniscal Extrusion Index), a measure of meniscal extrusion, that relates to various meniscal lesion patterns and has clinical and biomechanical significance. The study's secondary goal is to determine whether the standard 3 mm meniscal extrusion parameter still has value by correlating the MEI with it.

**Methods:**

1350 knee MRIs that were performed over the course of 2 years made up the study cohort. Following the application of inclusion and exclusion criteria, 200 of those patients were qualified to participate in the study. All the measurements examined for this study underwent an interobserver reliability test.

**Results:**

In the 1350 MRIs that were examined for this study, meniscal extrusion of any grade was present 18.9% of the time. The use of the MEI revealed three groups of patients: those with a MEI < 20%, who are likely para-physiological; those with a MEY between 20% and 40%, who are in a grey area; and those with a MEY > 40%, who have lesions that are impairing the proper meniscal function. According to the authors' findings, the percentage of meniscal extrusion did not correlate with the finite number (3 mm), making the 3 mm parameter an unreliable evaluation method.

**Conclusions:**

This study is clinically relevant, because it proposes a simple and reproducible classification of meniscal extrusion that may aid in evaluating the severity of an extrusion and help in the diagnosis of lesions that might be difficult to identify on MRI.

**Level of evidence:**

Level IV.

## Introduction

Meniscal extrusions (ME) refer to the complete or partial displacement of the meniscus beyond the tibial plateau and the tibial articular cartilage [15]. The biomechanical forces exerted on the knee during weight-bearing primarily impact the medial meniscus, resulting in extrusion forces or circumferential hoop stresses [12, 24]. Several anatomical structures play a crucial role in facilitating efficient medial meniscal function and preventing its extrusion [10, 21]. The most relevant structures are the meniscal roots, inter-meniscal ligaments, medial collateral ligament, and coronary ligament, which attaches to the medial side of the tibial plateau periphery [1, 25].

Meniscal posterior root tears (MPRT) and radial tears can result in the detachment of the meniscus from the tibial plateau, as well as the disruption of the circumferential fibers. These conditions compromise normal meniscus function and can lead to meniscal extrusion [6, 14].

The loss of functional capacity in an extruded meniscus can contribute to the progressive deterioration of osteoarthritis and the development of bone edema syndrome over time [2, 3, 13].

Degenerative meniscus lesions (DMLs) and complex tears, common in the over 50-year-old population, entail the degradation of collagen fibers in the meniscus. These conditions arise as a result of a complex interplay involving excessive use, mechanical overload, and impaired biological repair mechanisms [20].

Meniscal extrusions are evaluated with magnetic resonance imaging (MRI) and many categorizations have been proposed [22]; however, the most used classification distinguishes lesions as major (> 3 mm) and minor (< 3 mm) [4]. In addition, instead of a single value, other authors use in this study ranges of meniscal extrusion rates. Crema et al. classified medial and lateral meniscal extrusion as follows: no extrusion (grade 0), extrusion of 50% of the body (grade 1), and extrusion of > 50% of the body (grade 2) [5].

Miller et al. utilized a cutoff value of 25%, since this was the smallest amount they could confidently discern. They concluded that meniscal extrusion exceeding 25% of meniscal width was not significantly associated with a meniscal tear [19].

The aim of this study is to determine whether a percentage of meniscal extrusion, known as the Meniscal Extrusion Index (MEI), correlates with a specific pattern of meniscal lesion. This study introduces the MEI as a novel parameter that can aid clinicians in accurately assessing the degree of meniscal extrusion and help diagnose lesions that may not be easily identifiable on MRI.

The hypothesis of this study is that there is a correlation between the MEI and the severity of the underlying meniscal lesion.

## Materials and methods

The study was performed at Istituto Ortopedico Gaetano Pini and approved by the local ethical committee (authorization number: Fondazione IRCCS Ca’ Granda Ospedale Maggiore Policlinico–Milano Area 2, Lombardia, Milan (n°394_2019bis, Milan, 08.05.2019). The study was conducted in two distinct phases. The first phase was a literature review in March 2023 using the Pubmed/Medline and Cochrane databases. The objective was to identify all existing classifications of meniscal extrusion that had been described in the literature. In the second phase, a retrospective evaluation was carried out using imaging data obtained at the study hospital. The evaluations were performed using the same 1.5 Tesla MRI machine and study protocol. The exclusion criteria for this study were age under 18 or sign of incomplete growth, signs of previous surgery, malignancies or local expanding lesions or fractures. All knee MRI performed between July 2021 and June 2022 were analyzed and only those eligible for the study were included.

The MRI images were evaluated by two different researchers who assessed the inclusion and exclusion criteria. In case of any doubts or uncertainties, a third, more experienced researcher was involved in the decision-making process. PD, STIR and T2 Fat-Sat sequences were analyzed on the coronal plane. The level considered was the plane that presented the biggest with of the tibial spines as described by Costa et al. [4].

**Fig. 1:**
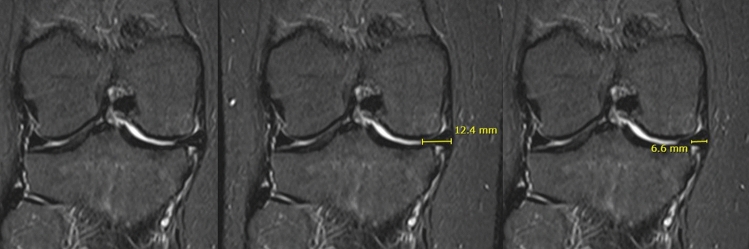
**A** Coronal MRI with a medial meniscal extrusion; **B** medial meniscal width; **C** overhanging portion of the meniscus

A meniscal extrusion (ME) was defined as any overhanging of the medial meniscal body over the tibial plateau on the analyzed plane. Osteophytes were not considered in this evaluation as that could alter the real tibial plateau width. The total medial meniscal width and the overhanging portion of the meniscus were measured, as described in Fig. 1.

The Meniscal Extrusion Index (MEI) was calculated by determining the ratio between the extruded portion of the meniscus and the total width of the meniscus. In the same imaging plane, the width of the medial tibial plateau and the width of the medial femoral condyle were also measured.

**Fig. 2:**
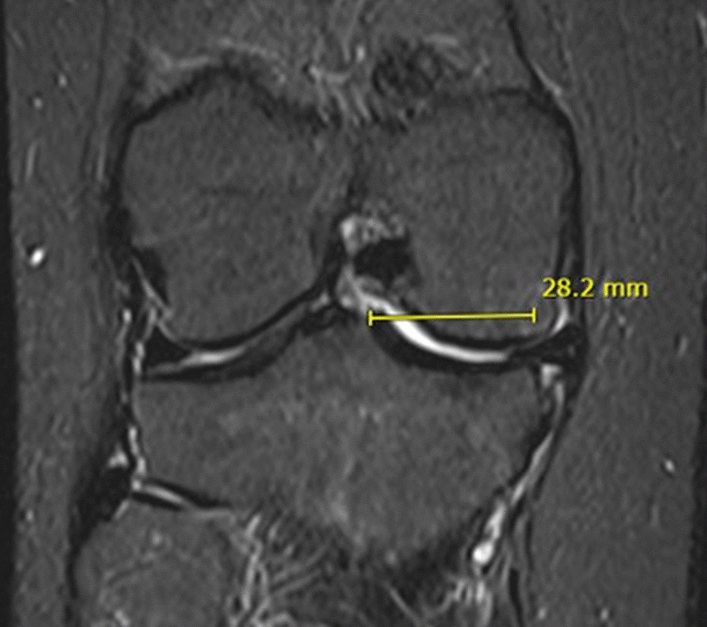
Medial tibial plateau width on Coronal MRI

The medial tibial plateau width was measured from the apex of the medial tibial spine parallel the tibial plateau up to the perpendicular to the medial tibial border, as shown in Fig. 2.

The medial femoral condyle width was performed in the same fashion between the lateral border of the medial condyle and the medial cortical bone. The level of the measured was selected as the end of the condyle curvature on the lateral side of the medial condyle.

The program used for the measurement was Xero Viewer, which allowed for the use of one decimal. The authors tried a different program that did not have this feature, but the results were unreliable due to the excessive approximation of the measurements.

The measurements of the meniscus, extrusion, tibial plate, and femoral condyle were recorded with one decimal place accuracy. For all other data, calculations were rounded to one decimal place.

Based on reports in the literature, all associated meniscal lesions were gathered and subdivided into four categories: lesions leading to extrusion, lesions that can lead to extrusion, lesions not leading to extrusion, and no lesions at all.

All evaluations were performed by two independent researchers and the interobserver reliability was then tested.

The inter-observer reliability and ICC analysis was interpreted according to Landis and Koch [17].

### Statistical analysis

Data were reported as median and interquartile range (IQR) when quantitative, and as counts and percentages when qualitative. Differences between quantitative data distributions were evaluated with the Wilcoxon test.

For both medial meniscal width, extruded meniscus, medial tibial plateau, and medial femoral condyle width inter-reader reproducibility were appraised via Bland–Altman analyses and reported as bias and coefficient of repeatability (CoR). Statistical analyses were conducted using Python 3.7.6, and *p* values < 0.05 were chosen as a threshold for statistical significance.

The sample size was calculated setting a significance level (threshold for *α*, or type 1, error) of 5% or 0.05, a desired power (1-*β*) of 80%, and estimating an effect size (*d*) of (M1–M2)/SD as per Cohen’s *d*, and a standard deviation of 0.05. As such, the d estimate would be 0.60, and according to the formula *n* = (*Z* * (sqrt(n1 + n2 + 1) + sqrt(n1)))^2^/(*d*^2^), the required sample size would have been 120 patients. All knee MRI performed between July 2021 and June 2022 were retrospectively retrieved.

## Results

A total of 1350 patients were eligible for MRI examinations, from 07/13/2021 to 05/30/2022. Meniscal extrusion was identified in 255 patients. A prevalence of 18.9% was, therefore, found in the population examined. After application of exclusions criteria, 55 exams were excluded from this study due to the presence of fractures, signs of previous surgery, tumors, low quality images or because the patients were under the age of 18. Two hundred medial meniscal extrusions were found to match the inclusion criteria.

Demographic data of the group of patients who received MRI assessment are reported in Table 1.

**Table 1:** Patients’ demographics

No. of patients	200
Age (years)	54.8 ± 14.2 57 [64–47]
Gender	
Female	112 (56)
Male	88 (44)
Side affected	
Left	94 (47)
Right	106 (53)

Data are reported as mean ± SD and median [Q1–Q3]

For each MRI was calculated the meniscal width (mm), the meniscal extrusion width (mm), the medial tibial plateau width (mm), and the medial femoral condyle width (mm). Results are reported in Table 2.

**Table 2:** Anatomical characteristics of the analyzed population

	Data
Meniscal width (mm)	11.1 ± 2.6 10.8 [12.4–9.4]
Meniscal extrusion width (mm)	4.2 ± 1.6 3.9 [5.0–2.9]
Medial tibial plateau width (mm)	30.2 ± 2.7 30.2 [32.2–28.0]
Medial femoral condyle width (mm)	30.1 ± 2.7 30.2 [32.2–28.0]

Data are reported as mean ± SD and median [Q1–Q3]

The variability of the meniscus’s width was 11.1 ± 2.6 Mean ± SD; 10.8 [12.4–9.4] Median [Q1–Q3].

**Fig. 3:**
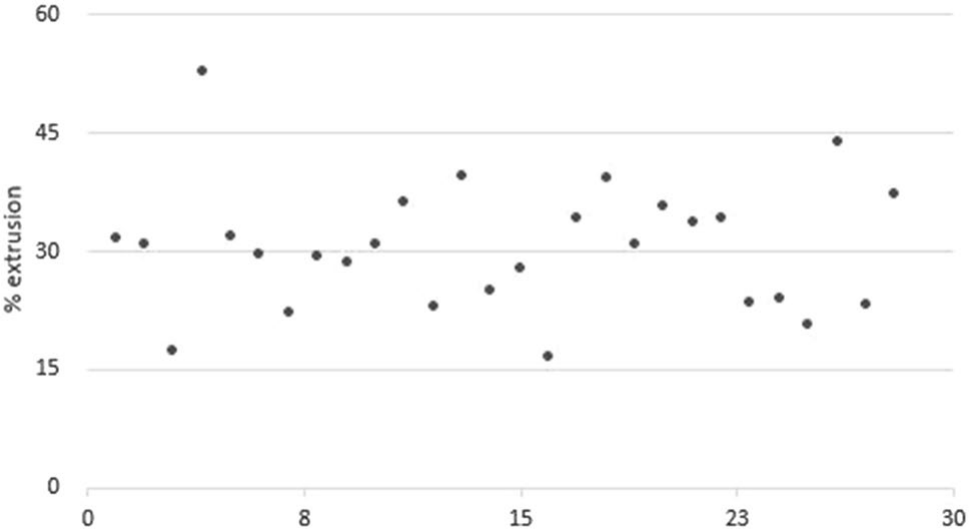
Dispersion of meniscal extrusion index (MEI) (%) in patients with Meniscal extrusion width (mm) of 3 ± 0.2 mm

Considering the meniscal extrusions of 3 ± 0.2 mm, their distribution was compared with the respective extrusion in % (MEI), as shown in Fig. 3.

All the MRI were categorized, based on the meniscal injury, in 4 groups: tears leading to extrusion (including Root lesions and radial tears), tears that might result in extrusion (complex tears and meniscal degeneration), tears not leading to extrusion (horizontal and longitudinal tears), no injuries (when no meniscal tear was found). The extrusion index was calculated for each group; the results are reported in Table 3.

**Table 3:** Meniscal Extrusion Index in the four study group

Group	Meniscal extrusion index (%)
Overall	39.2 ± 16.5 36.5 [49.9–26.4]
Tears leading to extrusion	49.8 ± 15.3 49.5 [57.0–38.2]
Tears that can lead to extrusion	42.4 ± 16.7 39.0 [53.5–29.6]
Tears not leading to extrusion	32.0 ± 12.7 30.2 [38.7–22.3]
No injuries	25.8 ± 7.8 23.5 [31.3–20.8]

Data are reported as mean ± SD and median [Q1–Q3]

**Fig. 4:**
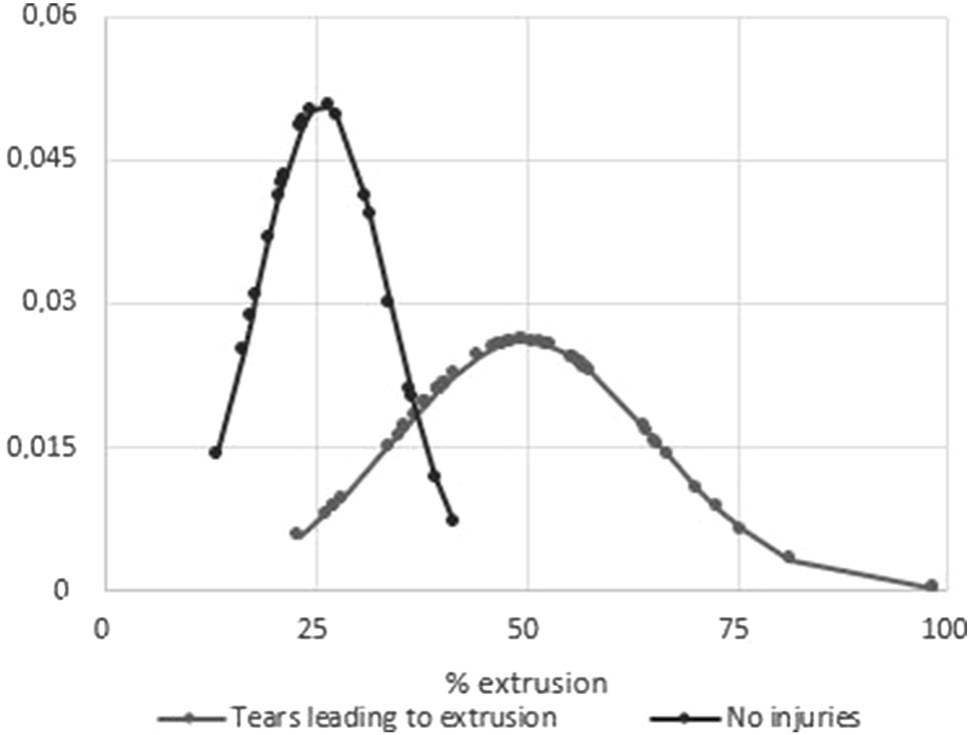
Correlation between the distribution of the extrusion index of the subpopulations Tears leading to extrusion and no injuries

In particular, the distribution of groups “tears leading to extrusion” and “no injuries” was reported and compared in Fig. 4.

Inter-observer reliability found an ICC of 0.996 which translate to an “almost perfect agreement”.

## Discussion

The most important finding of the present study was that the Meniscal Extrusion Index (MEI)-based classification of medial meniscal extrusion in the study showed a correlation with the severity of the underlying lesion. This classification system is simple, reproducible, and useful in clinical settings.

Abnormal medial meniscus extrusion is defined as protrusion of the body of the medial meniscus beyond the tibial plateau [15]. In literature the presence of a meniscal lesion is often associated with meniscal extrusion [7, 8, 23].

Due to the anatomy of the medial meniscus, various lesions are described as more or less responsible for meniscal extrusion [9, 11, 18].

Based on the available literature, the authors identified four distinct categories for meniscal lesions. The first group encompasses lesions that result in extrusion, such as root lesions and radial lesions. The second group consists of lesions that have the potential to cause extrusion, including complex tears and degenerative meniscal lesions. Group 3 comprises all lesions that do not lead to extrusion, such as horizontal and longitudinal tears. Finally, group 4 comprises patients without any meniscal lesions.

This study mainly focused on two groups: meniscal extrusions without any type of associated meniscal tear (group 4) and extrusions associated with lesions typically lead to extrusion (group 1).

The distribution of the two groups is illustrated in graph 2. Based on those data is evident that there are two very clear groups: patients with a MEI under 20% and patients with MEI over 40%.

In the first sub-group of patients, those with a MEI < 20%, there were no case of lesion known to be responsible for extrusion.

In the second sub-group (MEI > 40%) all patients presented an underlying lesion leading to extrusion.

Based on these data, a new classification of meniscal extrusions could be proposed, based on the MEI parameter:

- MEI smaller than 20%: slightly extruded, no underlying meniscal lesion causing functional insufficiency of the meniscus, probably para-physiological imaging feature.
- MEI between 20% and 40%: moderate extrusion, a gray area, high suspicion of an underlying lesion, unclear functional impairment.
- MEI higher than 40%: severely extruded menisci, always related to an underlying lesion that impairs the mechanical function of the meniscus.

The secondary outcome of this study was to evaluate if the standard 3 mm cut off proposed by Costa et al. was still viable. In their study, Costa et al. classified the degree of meniscal extrusion observed on imaging as major (> 3 mm) and minor (< 3 mm) [4].

In their research, they concluded that medial meniscus extrusion greater than 3 mm is significantly associated with extent of meniscal degeneration, extent of tear, complex tear, large radial tear, and tear involving the meniscal root.

In this study the authors analyze the measurement in mm of meniscal width, meniscal extrusion width, medial tibial plateau width, medial femoral condyle width. Results show that there is an individual variability of each parameter.

The authors found that there was no relationship between the finite number and the percentage of extrusion. The menisci extruded 3 ± 0.2 mm corresponded in the series analyzed to an extremely variable percentage of extrusion ranging from 52.8% to 16.5%.

The variability of the meniscus’s width found in this study makes the cutoff calculated in mm a limit.

For this reason, it would be more reliable to identify the extrusion as a percentage, expressed as mm of extruded meniscus compared to the total width of the meniscus.

Inter-observer reliability analysis performed confirms how this methodology is reproducible and how the MEI is easily calculated.

This study has some limitations, first being based on a retrospective sample.

The study group had a mean age of 54.8 years, so extrapolated data might not apply to a population that is young and athletic.

Moreover, while it can be assumed that all patients included in the MRI scans were experiencing symptoms, such as knee pain, the authors did not establish a correlation between the imaging findings and the patients' medical history or reported symptoms.

Basing data on MRI images, without arthroscopic confirmation, may be a limitation, although knee MRI has a documented high accuracy for diagnosis of medial meniscal tear, with sensitivities of 91.8% and specificities of 80.8% [9, 16].

The authors did not investigate additional extra-articular causes of meniscal extrusion mentioned in the literature. Factors such as knee misalignment, female sex, and high BMI were not considered in this study, as they are more likely to lead to intra-articular damage to the meniscus rather than being direct causes of extrusion.

Indeed, this study holds clinical relevance as it proposes a simple and reproducible classification system for meniscal extrusion. This classification can be valuable in assessing the severity of an extrusion and aiding in the diagnosis of lesions that may not be easily identifiable. By providing clinicians with a practical tool, this study contributes to improving the evaluation and understanding of meniscal extrusion in clinical practice.

## Conclusion

The study's classification of medial meniscal extrusion, based on the Meniscal Extrusion Index (MEI), demonstrates a correlation with the severity of the underlying lesion. This classification system is considered simple, reproducible, and applicable in clinical settings.

The study also highlights the potential for incorrect interpretation of the significance of meniscal extrusion when relying solely on the measurement in millimeters (mm). It demonstrates that the entity of meniscal extrusion can be over or underestimated using this approach.

## Data Availability

In accordance with the Italian d.lgs. 101/2018, data supporting Tables 1–3 and Figures 1–4 are not publicly available to preserve patient privacy. These datasets are available upon request from the corresponding author.
